# Effect of 1α,25-Dihydroxyvitamin D3 on the Radiation Response in Prostate Cancer: Association With IL-6 Signaling

**DOI:** 10.3389/fonc.2021.619365

**Published:** 2021-05-24

**Authors:** Chun-Te Wu, Yun-Ching Huang, Wen-Cheng Chen, Miao-Fen Chen

**Affiliations:** ^1^ Department of Urology, Chang Gung Memorial Hospital at KeeLung, KeeLung, Taiwan; ^2^ College of Medicine, Chang Gung University, Taoyuan, Taiwan; ^3^ Department of Urology, Chang Gung Memorial Hospital at Chiayi, Chiayi, Taiwan; ^4^ Department of Radiation Oncology, Chang Gung Memorial Hospital at Chiayi, Chiayi, Taiwan

**Keywords:** vitamin D3, IL-6, immune, radiation response, prostate cancer

## Abstract

Radiotherapy (RT) is the main treatment modality for prostate cancer (PCa). This study investigated the role of IL-6 in biological sequelae following irradiation and highlighted the effects of 1α,25-dihydroxyvitamin D3 (calcitriol) on the radiation response of PCa and its relationship with IL-6 signaling. Human and murine PCa cell lines were used to examine the response to irradiation *in vitro* and *in vivo*. The relationship of IL-6 expression with clinicopathologic characteristics in 104 PCa patients treated with definite RT was also examined. We also investigated the changes in radiation response after calcitriol supplementation and the relationship between calcitriol and IL-6 signaling by conducting cellular and animal experiments. Based on clinical samples, the positivity of IL-6 staining is a significant predictor of biochemical failure-free survival for PCa patients treated with definite RT. Data from preclinical models showed that inhibition of IL-6 increased the response of PCa to radiation, which was associated with increased oxidative DNA damage, attenuated EMT and MDSC recruitment, and decreased tumor regrowth. Moreover, increased vitamin D_3_ levels by calcitriol supplementation or induction by UVB-radiation was associated with inhibited IL-6 signaling and increased the response to irradiation observed in animal models. These data demonstrate that IL-6 play a critical role in the radiation response of PCa, which involved tumor cell killing and altering the tumor microenvironment. Directly targeting IL-6 signaling or vitamin D_3_ supplement with oral or light treatment could be a promising strategy to increase the response of PCa to radiation.

## Introduction

In prostate cancer (PCa), radiotherapy (RT) is one of the main treatment modalities to reduce disease recurrence. Despite progress in the delivery modalities, post-RT relapse still occurs in a fraction of treated patients. A better understanding of the factors regulating the radiation response of PCa is an important issue.

The tumor-promoting activities of inflammation have been widely studied (1). Furthermore, several inflammatory cytokines are believed to play key roles in the radiation response and thought to lead to tumor promotion and recurrence (2). Irradiation induces upregulation of a variety of cytokines, such as IL-6. IL-6 expression is reported to be increase in a variety of tumors and to contribute to aggressive tumor growth and resistance to treatment (3, 4). In patients with high IL-6 levels, the response to therapy was worse (5–7). Moreover, IL-6/STAT3 activation has been reported to mediate the radioresistance of tumors (8–10). We previously reported that overexpressed IL-6 and activated STAT3 signaling mediate the radioresistance of hormone-refractory (HR) PCa (9). Therefore, the role of IL-6 signaling in the radiation response of PCa was further investigated in the present study.

Evidence shows that vitamin D deficiency is associated with increased incidences of cancer and impacts the treatment response (11–13). The most biologically active form of vitamin D, namely, 1α, 25(OH)_2_ D_3_ (calcitriol), exerts potent anticancer and anti-inflammatory effects in cells by cell- and tissue-specific manner (11, 14). Calcitriol was reported to induce cellular arrest, trigger apoptotic signaling, and regulate reactive oxidative stress (ROS) and inflammation to affect cancer development and growth. In addition, calcitriol was shown to augment the tumor inhibition induced by anticancer modalities, including RT (15, 16). Calcitriol shows actions in several pathways, including the p38-MAPK, IL-6, TGF-β1 and NF-κB signaling pathways. Although vitamin D3 had antitumor abilities noted in preclinical cancer models, the role of vitamin D3 in the radiation response of PCa and the molecular mechanism involved need further investigation. We proposed that calcitriol has the potential to regulate IL-6 signaling. Based on this hypothesis, we examined the link among vitamin D3, IL-6, and radiation response and the mechanisms of action for PCa in this study.

## Materials and Methods

The study protocol was approved by the institutional review board of Chang Gung Memorial hospital (No. 201701798B0). We enrolled 104 patients who had histologically confirmed PCa and received curative-intent RT at our hospital between Jan 2005 and Dec 2014. Needle core biopsy specimens collected from PCa patients at diagnosis were subjected to immunochemical analyses. Patients who received RT with ≥ 70 Gy were included in this retrospective study. The clinical characteristics of the patients are shown in **Table 1**.

**Table 1:** Clinical characteristics of patients.

	No. of patients		*p* value
	IL6 (-)	IL6 (+)	
**Number**	63	41	
**Age**			0.42
Median	71.2	69.6	
Range	48.4-83.5	47.6-83.3	
**Stage**			<0.001^*^
T1-T2N0	48	10	
Advanced stage^a^	15	31	
**Gleason score**			0.013^*^
<7	34	12	
>=7	29	29	
**Pre-Tx PSA**			<0.001^*^
<20	40	10	
>=20	23	31	
**NLR**			<0.001^*^
<3	37	10	
>=3	9	23	
Unknown	17	8	
**Biochemical failure**			<0.001^*^
Negative	56	24	
Positive	7	17	
**Survival status**			0.722
Alive	51	32	
Dead	12	9	

a stage T3-T4 or regional lymph node involvement. *means statistical significance.

### Statistical Analysis

The main end point was biochemical failure and biochemical failure-free survival (time elapsed between diagnosis and biochemical failure or death from any cause). We used the Kaplan-Meier method for survival curves, and the log-rank test to determine differences between groups. Samples were analyzed using Student’s t-test. Data are presented as the mean ± standard error of the mean (SD). Each experiment was performed independently at least twice. A probability level of p < 0.05 was taken to indicate statistical significance, unless otherwise stated.

### Immunohistochemical Staining

The core from the tumor with the highest Gleason sum or, in case of only one Gleason pattern, from the largest tumor was subjected to IHC staining. The sections were then incubated overnight with anti-IL-6 antibody (1:20). The staining patterns on slides containing 5-6 core biopsy specimens per patient were assessed using a semiquantitative immunoreactive score (IRS), as previously described. All tumor cells on the slides were evaluated. An IRS scoring grade of >= 2 was considered a positive IHC score.

### Mouse Tumor Models (Ectopic and Orthotopic)

Eight-week-old male C57BL/6J and nude mice were used as tumor implantation model mice. All mouse experiments were approved by the experimental animal committee of our hospital (N0. 20170112901); the details were described previously (8, 9, 17). Briefly, transfected 22RV1 and TRAMP-C1 cells were implanted subcutaneously in the ectopic tumor implantation model and intraoperatively into the lateral region of the prostate gland in the orthotopic tumor implantation model. To determine radiosensitivity *in vivo*, local radiation (15 Gy) was performed when the tumors reached 0.5 cm3 in volume (ectopic model) or 2 weeks after implantation (orthotopic model). The effects of calcitriol on tumor radiosensitivity were also investigated *in vivo*. In the treatment groups, intraperitoneal injection of calcitriol (0.5 μg/kg per mouse) was started 1 day before radiation and continued as scheduled. We also examined the role of ultraviolet B (UVB) radiation *in vivo*. UVB light-emitting diodes (300 nm) were obtained from the Instrument Technology Research Institute (Hsinchu, Taiwan). In the UVB-treated groups, treatment was initiated on the day of tumor implantation and was performed for 30 minutes per day, five times per week.

### Flow Cytometric Analyses of Myeloid-Derived Suppressor Cells (MDSCs)

As described previously (8, 9), FACS was conducted with single-cell suspensions prepared from murine spleen to count the numbers of mouse MDSCs, which were identified as CD11b+Gr1+ with fluorescence-labeled monoclonal antibodies (BD PharMingen).

### Cell Culture and Reagents

The TRAMP-C1 cell line, which was derived from a prostate tumor of C57BL/6 mice (transgenic adenocarcinoma of the mouse prostate (TRAMP)), was cultured and stably transfected with control vector (CV) or IL-6-silencing vector (SV). We also cultured human 22RV1 cells as described earlier (18). The cytokine IL-6, IL-6-neutralizing antibody and calcitriol were purchased from R&D Systems (Minneapolis, MN, USA) and Sigma (St. Louis, MO, USA), respectively. Cancer cells with stable silencing of IL-6 were generated by transfecting TRAMP-C1 and 22RV1 cells with the SV and selected by culturing in medium containing puromycin for 4 weeks (19). To determine the *in vitro* effects of calcitriol, protein analysis was performed for cells incubated in the presence of the indicated dosage of calcitriol for 48 hours (20).

### Clonogenic Assays

To determine intrinsic cellular radiosensitivities, clonogenic assays were performed. Exponentially growing cells were irradiated and incubated for 10 days at 37°C. The plates were stained with crystal violet (Sigma) to aid colony counting. Colonies containing >50 cells were scored to determine plating efficiency and the fractions of the cells surviving after application of the various treatments.

## Results

### Role of IL-6 in the Response of PCa to Radiation In Vitro

**Figure 1:**
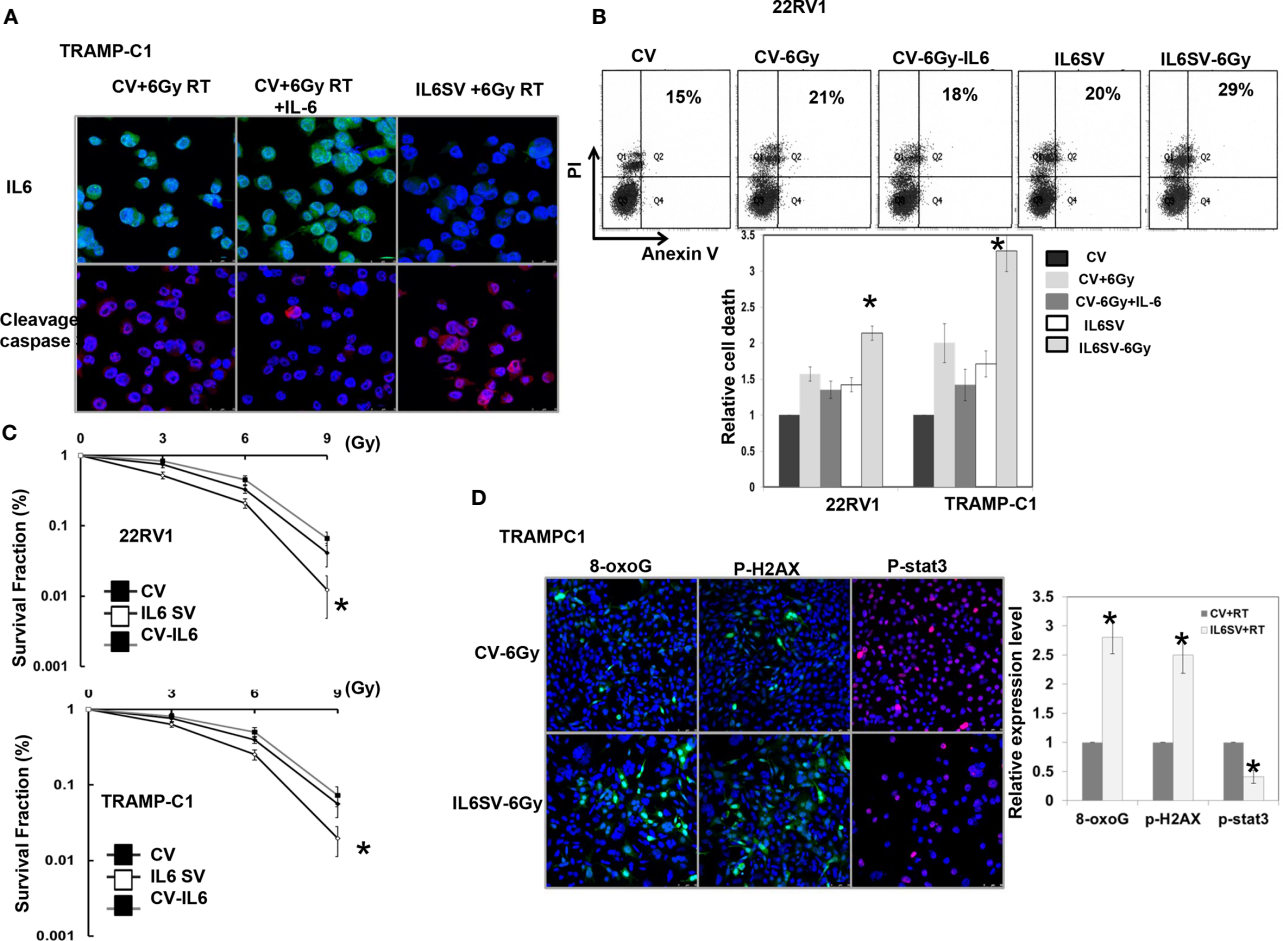
Effects of IL6 on radiation responses *in vitro.* **(A)** IL6 silencing vectors significantly decreased IL6 expression associated with increased apoptosis-related proteins by immunofluorescence *in vitro* (DAPI, blue; Cleavage caspase 3, red; IL-6, green). **(B)** The *in vitro* effects of IL6 on apoptosis and cell death, as evaluated by FACS with Annexin V-PI staining in irradiated cells. The data revealed that IL-6 inhibition increased apoptosis in irradiated cells. **(C)** Cells were irradiated with 0, 3, 6, or 9 Gy, and the survival curves were determined by colony-formation assay. The survival fraction was determined by enumerating colonies after irradiation exposure divided by the plating efficiency of the respective cells. Points, means of 3 separate experiments; bars, SD. *P < 0.05. (CV: prostate cancer cells transfected with control vectors; IL6 SV: prostate cancer cells transfected with IL-6 silencing vectors) **(D)** IL6 silencing vectors significantly decreased activated stat3 expression associated with increased DNA damage after RT, as demonstrated by immunofluorescence *in vitro*. The quantification is to calculate the value of the cell number positive for targeted protein divided by the total cell number. The Y-axis represents the ratio normalized by the value of irradiated CV cells. *p < 0.05. (CV: prostate cancer cells transfected with control vectors; IL6 SV: prostate cancer cells transfected with IL-6 silencing vectors; cell-6Gy: transfections with 6Gy irradiation; CV-IL-6: CV cells were treated with IL6 10ng/ml for 3 days before irradiation).

To determine the role of IL-6 in the radiosensitivity of PCa, TRAMP-C1 and 22RV1 cells were transfected with IL-6 SV or CV. Cells transfected with IL-6 SV significantly inhibited IL-6 expression (**Figure 1A**) compared to that of cells transfected with CV. As shown in **Figures 1A, B**, blocking IL-6 increased the expression of cell death- and apoptosis-related proteins induced by RT. We also examined the sensitivity of PCa cell lines to radiation by clonogenic assay. Cells were exposed to single radiation doses of 0, 3, 6 or 9 Gy, and their survival curve was determined and normalized to the plating efficiency of the respective cells. **Figures 1C, D** revealed that blocking IL-6 increased the RT-induced loss of clonogenic cells, which was associated with attenuated STAT3 activation and increased oxidative DNA lesions. Furthermore, the IL-6 SV increased the expression of p-H2AX, which is linked to the formation of DNA double-strand breaks induced by RT.

### Role of IL-6 in the Response of PCa to Radiation *In Vivo*

**Figure 2:**
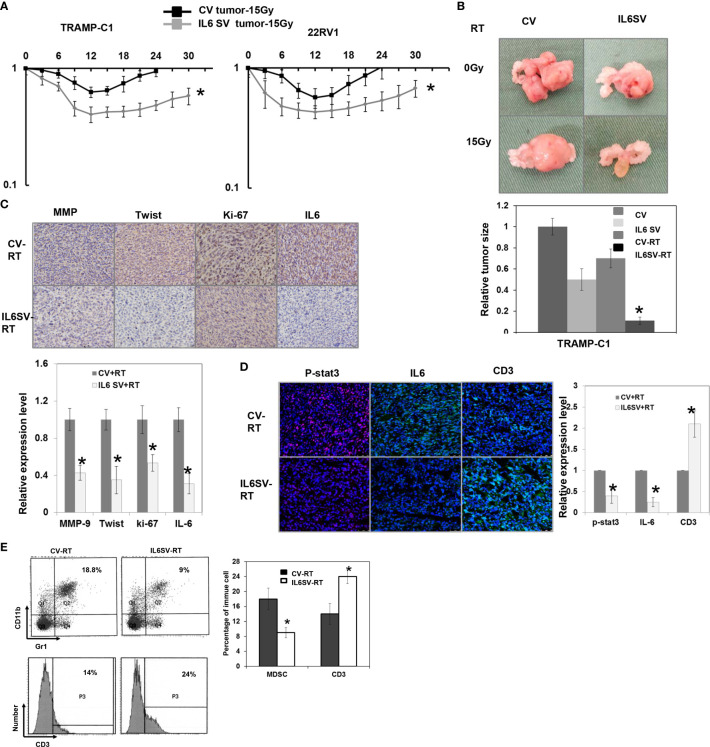
Effects of IL6 on radiation responses *in vivo.* **(A)** The effect of IL-6 on the radiosensitivity of prostate cancer (22RV1 and TRAMP-C1), as demonstrated by tumor growth delay of the ectopic tumor after 15 Gy irradiation (CV: cells transfected with control vectors; IL6 SV: cells transfected with IL6 silencing vectors), and **(B)** The relative tumor size of orthotopic tumor at 12 days after 15Gy irradiation or sham irradiation. The relative tumor size was normalized to the CV tumor size 12 days after sham irradiation (CV-RT: orthotopic tumor of cells transfected with control vectors after 15Gy irradiation; IL6 SV-RT: orthotopic tumor of cells transfected with IL6 silencing vector after 15Gy irradiation). **(C)** The effect of IL6 SV on tumor regrowth after irradiation is demonstrated by IHC using Ki-67, MMP-9, and twist staining (CV-RT: tumor of cells transfected with control vectors after 15Gy irradiation; IL6 SV-RT: tumor of cells transfected with IL6 silencing vector after 15Gy irradiation), and **(D)** the tumor infiltrating CD3-positive T cells by immunofluorescence *in vivo* (DAPI, blue; p-STAT3, red; IL-6/CD3, green). Representative images are shown. The quantification is to calculate the value of the cell number positive for targeted protein divided by the total cell number. The Y-axis represents the ratio normalized by the value of irradiated CV cells. *p < 0.05. **(E)** IL6 inhibition was associated with attenuated myeloid-derived suppressor cells (MDSC) recruitment and increased infiltration of CD3+ T cells in tumors by FACS.

The analysis of human PCa cells in immunocompromised mice (**Figure 2A**) confirmed the *in vitro* findings to show that the SV increased the responsiveness of PCa to RT. To investigate the role of IL-6 in the immune tumor microenvironment and the response of PCa to radiation, we implemented ectopic and orthotopic tumor animal models in immunocompetent mice. **Figures 2A, B** shows that TRAMP-C1-derived prostate tumors with inhibited IL-6 expression had a longer delay of tumor growth of subcutaneous tumors and smaller orthotopic tumors following RT. Moreover, **Figures 2C, D** shows that blocking IL-6 attenuated the expression of Ki-67, a marker of cell proliferation, and changes in epithelial-to-mesenchymal transition (EMT) in regrowing tumors after RT. Cumulative experimental evidence indicates that the irradiated tumor microenvironment, including immune cells and the inflammatory response, contributes to the radiation response. It was assumed that MDSC recruitment plays a role in tumor regrowth after radiation (21). MDSCs are characterized by their ability to suppress T cell functions, including proliferation (22); therefore, we further examined the activation of MDSCs and T cell infiltration in irradiated prostate tumors. The FACS and immunofluorescence analyses demonstrated that radiation stimulated MDSC recruitment. As shown in Fig. 2e, blocking IL-6 was associated with attenuated MDSC recruitment and increased T cell infiltration in irradiated tumors.

### Role of IL-6 in Patients With Prostate Cancer

**Figure 3:**
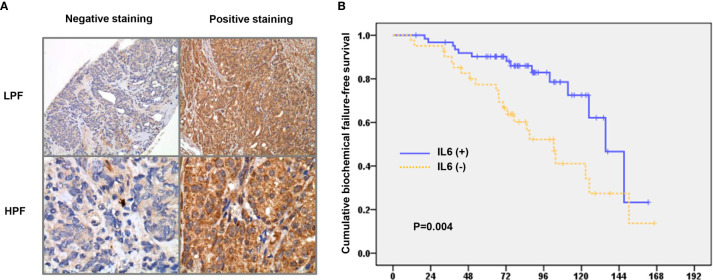
Role of IL-6 in prostate cancer patients treated with definite radiotherapy. **(A)** Representative slides of tumor specimens positively or negative staining for IL-6 are shown (LPF, lower power field; HPF, high power field). **(B)** The differences of biochemical failure-free survival according to the staining of IL-6.

**Figure 3A** shows representative slides with positive and negative IHC staining for IL-6 in human cancer specimens at diagnosis. Of the 104 PCa patients, 39 (40%) showed overexpression of IL-6. As shown in **Figure 3B** and **Table 1**, positive staining for IL-6 was significantly associated with an advanced clinical stage, higher PSA level, and elevated Gleason score. Furthermore, based on the survival analysis, both clinical stage and IL-6 level had predictive power with regard to biochemical failure in PCa patients. These findings suggest that IL-6 could predict the radiation response and prognosis of PCa patients treated with definite RT.

### Effects of Calcitriol on the Response of PCa to Radiation *In Vitro*

**Figure 4:**
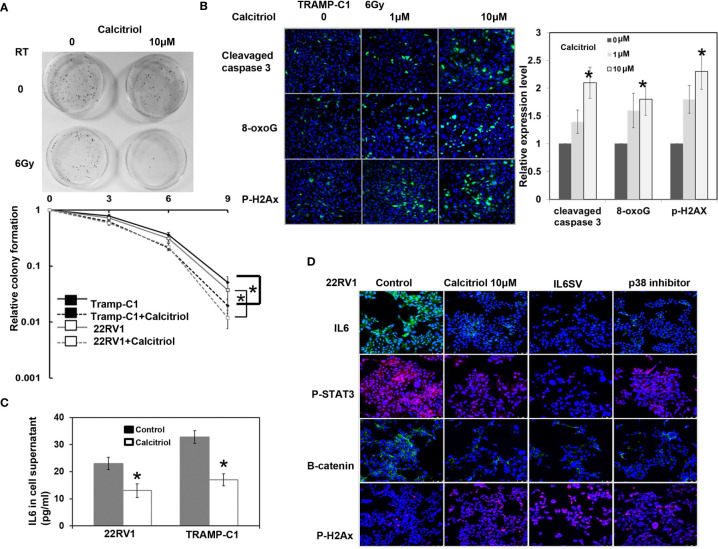
Effects of Calcitriol on radiation responses *in vitro*. ** (A)** Calcitriol significantly increased RT-induced cell death by clonogenic assay. The survival fraction was determined by enumerating colonies after irradiation exposure divided by the colony numbers with the respective condition and sham irradiation. Points, means of 3 separate experiments; bars, SD. *P < 0.05. **(B)** Effect of Calcitriol treatment on apoptosis-related protein and DNA damage was demonstrated by immunofluorescence *in vitro* (DAPI, blue; target protein, green). The quantification is to calculate the value of the cell number positive for targeted protein divided by the total cell number. The Y-axis represents the ratio normalized by the value of irradiated cells under control condition. *p < 0.05. **(C)** The levels of IL-6 in cellular supernatants were examined by ELISA *in vitro*. Columns represent the means ± SD. *P < 0.05. **(D)** The expression levels of IL-6/p-STAT3, B-catenin and Ki-67 were evaluated by immunofluorescence using 22RV1 cells incubated in the presence of indicated dosage of calcitriol or p38 inhibitor for 48 h and the results of representative slides are shown.

As shown by a colony formation assay over a one-week period, **Figures 4A, B** and **Supplemental Figure 1** revealed that calcitriol increased the loss of clonogenic cells, which was associated with increased RT-induced apoptosis and DNA damage and attenuation of EMT changes. Calcitriol was also found to inhibit IL-6 expression and STAT3 activation in both 22RV1 and TRAMP-C1 PCa cells (**Figures 4C, D** and **Supplemental Figure 2**). Inhibition of p38 signaling has been suggested to be mediated by some of the activities of vitamin D in cancer (23). As indicated by protein analysis *in vitro*, calcitriol treatment inhibited p38 phosphorylation in cells (Suppl. Fig. 2). Furthermore, as shown in **Figure 4D**, calcitriol-induced inhibition of IL-6 signaling was similar to that elicited by the p38 MAPK inhibitor *in vitro*. Therefore, we suggest that the inhibited IL-6 signaling induced by calcitriol might be mediated at least partially by attenuating p38 signaling.

### Effects of Calcitriol on the Response of PCa to Radiation *In Vivo*

**Figure 5:**
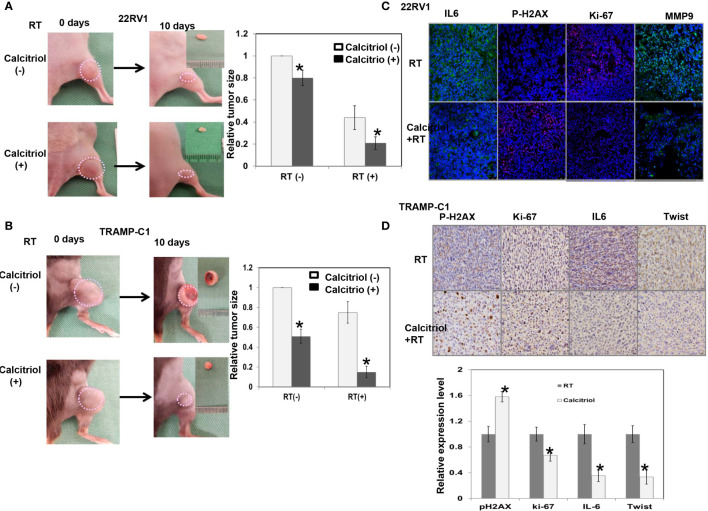
Effects of calcitriol on the radiation sensitivity *in vivo*. **(A)** The effect of calcitriol on the radiosensitivity of prostate cancer (22RV1) in immunocompromised mice, and **(B)** prostate cancer (TRAMP-C1) in immunocompetent mice, as demonstrated by relative tumor size of the ectopic tumor. Representative images and quantitative data are shown indicated days after 15 Gy radiotherapy (RT). The y axis represents the relative ratio, normalized to the tumor size of 22RV1 and TRAMP-C1 tumors under sham irradiation, respectively. **(C)** The effect of calcitriol on tumor after irradiation is demonstrated by immunofluorescence using IL-6, Ki-67, MMP-9, and p-H2AX staining in 22RV1 tumor (DAPI, blue; Ki-67/p-H2AX, red; IL-6/MMP-9, green), and **(D)** IHC using IL-6, Ki-67, Twist, and p-H2AX staining in TRAMP-C1 tumor. Representative images are shown. The quantification is to calculate the value of the cell number positive for targeted protein divided by the total cell number. The Y-axis represents the ratio normalized by the value of irradiated tumor without calcitriol treatment. *p < 0.05.

The data of human PCa cells in immunocompromised mice (**Figure 5A**) showed that calcitriol supplementation increased the responsiveness of PCa to radiation, as demonstrated by increased RT-induced tumor regression. We also examined ectopic tumor animal models in immunocompetent mice to investigate the role of calcitriol in the response of PCa to radiation and the link between calcitriol and the immune tumor microenvironment. **Figure 5B** shows that calcitriol significantly increased RT response, as demonstrated by the reduction in the relative size of subcutaneous tumors after radiation. Moreover, **Figures 5C, D** shows that calcitriol attenuated IL-6 signaling, the expression of cellular proliferation markers and EMT changes and increased DNA damage in radiated tumors.

### UVB Treatment Enhances the Effects of Calcitriol on Reducing IL-6 Production and Increasing the RT Response *In Vivo*

**Figure 6:**
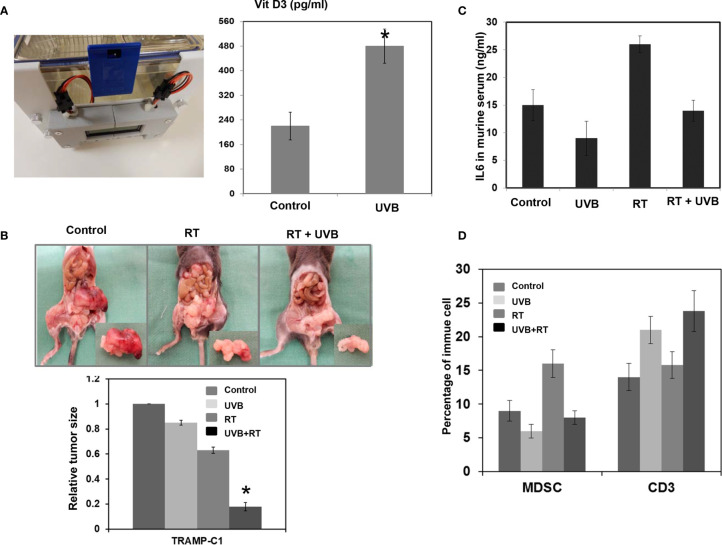
UVB treatment reduces IL-6 production and increase the radiosensitivity of prostate cancer. **(A)** UVB light treatment for 5 days increased calcitriol serum levels in C57/BL6 mice. Sham irradiation was performed as a control. **(B)** The representative picture of orthotopic tumor at 12 days after 15Gy irradiation with or without UVB treatment. The relative tumor size was normalized to the TRMP-C1 tumor size 12 days after sham irradiation. **(C)** The levels of IL-6 in murine were examined by ELISA in mice. Columns represent the means ± SD. *P < 0.05. **(D)** UVB treatment was associated with attenuated myeloid-derived suppressor cells (MDSC) recruitment and increased infiltration of CD3+ T cells in tumors by FACS.

The concentrations of calcitriol required for its antineoplastic effects are usually high; these high levels are difficult to achieve by daily supplementation with vitamin D_3_ alone. Here, we examined the ability of the UVB light treatment on the increase of calcitriol and the enhancement of RT response. UVB light treatment in mice led to profoundly increased serum calcitriol levels (**Figure 6A**). As shown in **Figures 6B–D**, UVB light treatment significantly suppressed PCa growth following RT and was associated with reduced IL-6 serum levels and diminished MDSC recruitment.

## Discussion

IL-6 is an important factor that regulates oncogenic signaling and is correlated with resistance to cancer treatment (24, 25). The radiation response was evaluated using assays that consider various types of radiation-induced cell death, specifically, *in vitro* clonogenic assays and *in vivo* tumor size measurements. Our data indicate that IL-6 expression is linked with*in vitro* and *in vivo* biological changes in PCa following irradiation. When IL-6 was inhibited by the SV, RT-induced cell death of PCa cells was increased; this was associated with increases in RT-induced p-H2A.X expression and oxidative DNA damage. Tumor relapse after RT is substantially affected by systemic factors and depends on the features of the tumor microenvironment (21, 22, 26). EMT is reported to be associated with tumor invasion and radioresistance (26–28). IL-6/STAT3 signaling was reported to stimulate tumor invasion, EMT and promote the survival of tumor cells after therapy to acquire treatment resistance (9, 29). Based on these data, we suggest that in addition to increased cell death, an attenuation in EMT contributes, in part, to the increased response to RT induced by IL-6 inhibition. MDSCs, an important subset of cells that contribute to an immunosuppressive tumor microenvironment, are reported to facilitate tumor regrowth after irradiation (2, 22, 30). Our data demonstrated that there was a positive correlation between the MDSC subpopulation and IL-6 levels in mouse tumors. The IL-6 SV significantly reduced MDSC activation and increased the number of infiltrating T cells. The role of IL-6 in PCa was further investigated by analyzing clinical specimens. Positive IL-6 staining was significantly associated with a more advanced clinical stage, elevated PSA levels, and a higher Gleason score. Furthermore, the survival analysis revealed that overexpression of IL-6 predicted biochemical failure in PCa patients. The correlation of IL-6 and the risk of biochemical failure revealed that IL-6 positivity correlated with the radiation response of PCa, suggesting that IL-6 is critical in this response.

Much of the current interest in combining immunomodulation and RT lies in strategies to overcome the tumor-promoting microenvironment. It has been reported that vitamin D3 has a potential role in modulating local and systemic immune responses and affecting the RT response (31–34). Vitamin D3 or its analog has been shown to enhance the response of breast cancer cells and PCa cells to radiation, stimulate immune infiltration and decrease the levels of anti-inflammatory cytokines to increase immune control of the tumor. We investigated the effect of calcitriol on the radiation response in PCa and found that calcitriol increased RT-induced cell death and augmented DNA damage associated with a greater decrease in tumor size following RT. Furthermore, calcitriol inhibited IL-6 expression and EMT changes in irradiated cancer cells. In tumor-bearing mice, calcitriol attenuated MDSC recruitment and increased the numbers of cytotoxic T cells at the tumor site after irradiation. Furthermore, Vitamin D_3_ exhibits several anti-inflammatory effects, including inhibition of p38 stress kinase signaling and the subsequent production of proinflammatory cytokines in multiple cancers that likely contributes to its beneficial effects (11, 31, 35). As shown through protein analysis *in vitro* and *in vivo*, calcitriol treatment significantly increased the radiation response, decreased the levels of p-p38 associated with attenuated IL-6 signaling. Moreover, calcitriol-mediated inhibition of IL-6 signaling was similar to that induced by the p38 MAPK inhibitor. Therefore, we suggest that the inhibition of p38 phosphorylation is the underlying mechanism that is at least partially responsible for calcitriol-induced suppression of IL-6 signaling in PCa. Although calcitriol could be obtained from dietary sources or produced by exposure to sunlight, the concentrations required for the antineoplastic effects of calcitriol are supraphysiological and may induce hypercalcemia, which can be a major problem men with PCa. Therefore, we applied UVB light to increase vitamin D3 serum levels in mice. In C57BL/6 mice, UVB light treatment for 30 minutes per day (5 days per week) significantly increased calcitriol serum levels and reduced xenograft tumor growth following RT. Additionally, UVB light treatment was found to reduce IL-6 levels and attenuate MDSC recruitment in tumor-bearing mice following RT.

## Conclusion

Based on the data in this study, we suggest that IL-6 signaling plays a critical role in the radiation response in PCa. Moreover, calcitriol supplementation *via* oral or light treatment is a promising strategy to increase the RT response in PCa, especially for patients with IL-6-positive PCa.

## Data Availability Statement

The raw data supporting the conclusions of this article will be made available by the authors, without undue reservation.

## Ethics Statement

The studies involving human participants were reviewed and approved by Chang Gung Memorial Hospital. The patients/participants provided their written informed consent to participate in this study. The animal study was reviewed and approved by Chang Gung Memorial Hospital.

## Author Contributions

C-TW participated in its design and statistical analysis, performed the study, and drafted the manuscript. Y-CH and W-CC conceived of the study and participated in its design and coordination. M-FC participated in its design and statistical analysis, performed the study, and coordination and assisted in editing of manuscript. All authors contributed to the article and approved the submitted version.

## Funding

The work was support by Chang Gung Memorial Hospital. Grant CMRPG6H0223 (to M-FC), and Chang Gung Memorial Hospital. Grant CMRPG6D0221-3 (to C-TW).

## Conflict of Interest

The authors declare that the research was conducted in the absence of any commercial or financial relationships that could be construed as a potential conflict of interest.
