# Supplementary material for: Effect of 1α,25-Dihydroxyvitamin D3 on the Radiation Response in Prostate Cancer: Association With IL-6 Signaling
Source: Front Oncol. 2021 May 24;11:619365. doi: 10.3389/fonc.2021.619365 (PMC8181126; doi:10.3389/fonc.2021.619365)

Suppl. Figure Legends

Figure 1 Effects of Calcitriol on radiation responses *in vitro*

Effect of Calcitriol treatment on EMT-related proteins demonstrated by immunofluoresence *in vitro* (DAPI, blue; target protein, green).

Figure 2 Effects of Calcitriol on IL6 signaling *in vitro*

Effect of Calcitriol treatment on IL6 expression demonstrated by immunofluoresence *in vitro* (DAPI, blue; IL-6, green; p-stat3/p-p38, red).

Suppl. Figure 1

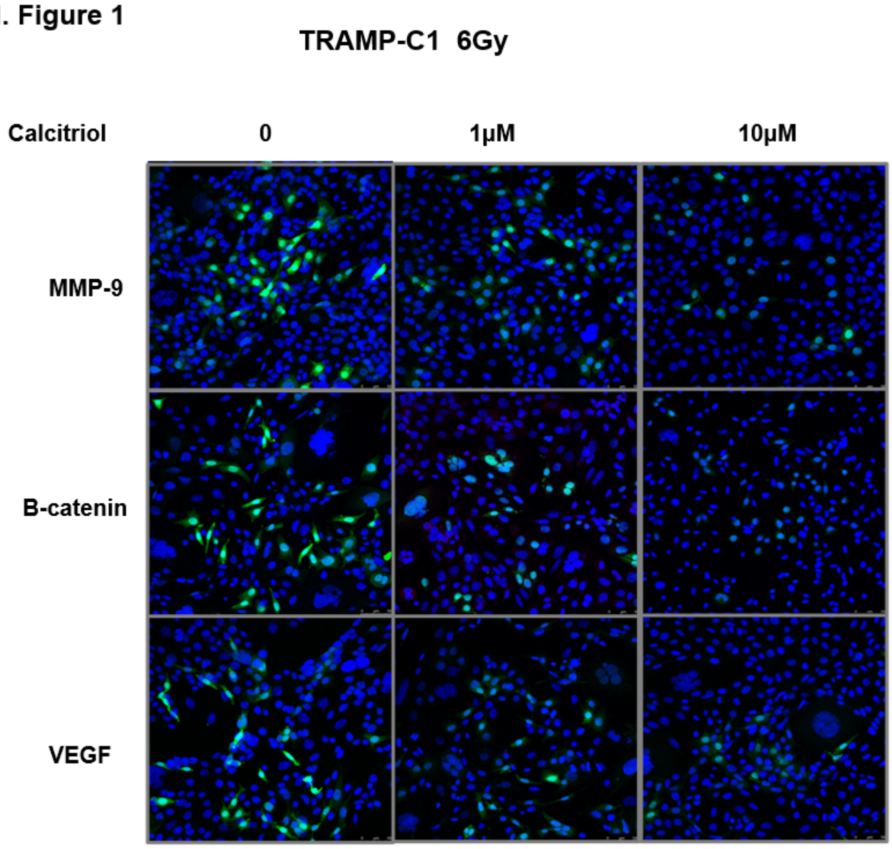

Suppl. Figure 2

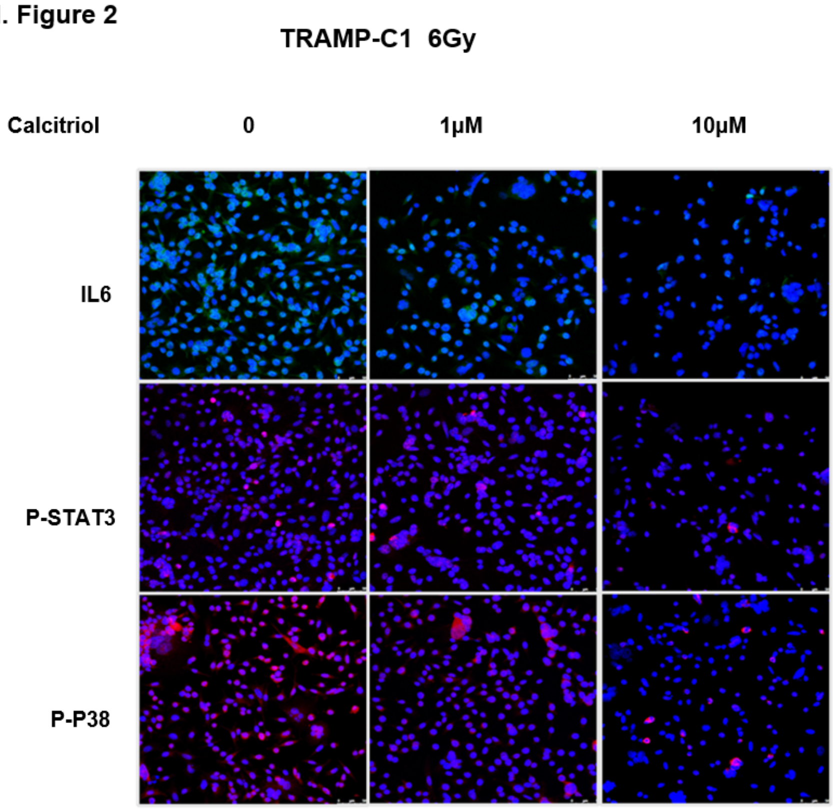

Supplement: Supplementary file 1 [file DataSheet_1.pdf]
